# Complete genome sequence of *Halomonas* sp. strain M1, a thiosulfate-oxidizing bacterium isolated from a hyperalkaline serpentinizing system, Ney Springs

**DOI:** 10.1128/MRA.00508-23

**Published:** 2023-10-31

**Authors:** Mehdi Alibaglouei, Leah R. Trutschel, Annette R. Rowe, Joshua D. Sackett

**Affiliations:** 1 Department of Biological Sciences, University of Cincinnati, Cincinnati, Ohio, USA; Montana State University, Bozeman, Montana, USA

**Keywords:** heterotrophic sulfur oxidation, alkaliphile, serpentinization, thiosulfate oxidation

## Abstract

We report the full genome sequence of *Halomonas* sp. strain M1, isolated from a continental high pH serpentinizing spring in northern California, USA. The 3.7 Mb genome has a G + C content of 54.13%, encodes 3,354 protein-coding genes, and provides insights into the metabolic potential for sulfur oxidation.

## ANNOUNCEMENT

Serpentinizing systems are hyperalkaline environments often characterized by high concentrations of hydrogen and/or methane (1 – 3). Thermodynamic, metagenomic, and activity assessments have demonstrated the capacity for various redox-active sulfur species to serve as electron donors or acceptors in many of these systems (4, 5). Here, we present the complete genome sequence of *Halomonas* sp. strain M1, a chemolithoheterotrophic thiosulfate-oxidizing bacterium isolated from Ney Springs. Ney Springs is a hyperalkaline (pH >12) marine-like terrestrial serpentinizing system characterized by high methane, ammonia, sulfide, and thiosulfate concentrations and abundant putative lithoheterotrophs, including *Halomonas* species (6).

Ney Springs is located near Mt. Shasta, California, USA (41°16′14.0′′N, 122°19′27.3′′W). M1 was isolated from Ney Springs cistern scrapings aseptically collected below the water surface and grown aerobically on solid Ney Springs minimal media (10 mM acetate, 15 mM thiosulfate, 22°C–25°C) as previously described (6). Media preparation instructions are available here: dx.doi.org/10.17504/protocols.io.bqjgmujw (7). M1 was grown aerobically in liquid minimal media (10 mM acetate, 20 mM thiosulfate) at 22°C–25°C for 4 days without shaking. DNA was isolated from a cell pellet using the Qiagen DNeasy PowerSoil Kit (Germantown, MD) and quantified via Qubit (ThermoFisher Scientific, Waltham, MA). Illumina and Nanopore libraries were prepared from the same DNA prep. Illumina libraries were prepared with an Illumina DNA Prep Kit (San Diego, CA), barcoded with 10 bp unique dual indexing (UDI) indices, and sequenced on an Illumina NovaSeq (2 × 150 sequencing chemistry) at SeqCenter (Pittsburgh, PA). Oxford Nanopore MinION sequencing libraries were prepared using the Native Barcoding Kit 24 V14 Kit (Oxford Nanopore Technologies, Oxford, UK) and sequenced on a R10.4.1 flow cell (FLO-MIN114) under high-accuracy mode (280 bp/s). Basecalling was performed using Guppy 6.4.6, and low-quality reads (quality score <7) were removed. Filtlong 0.2.1 (8) was used to quality filter Nanopore sequences, discarding reads <2 kb and the worst 10% of read bases. Nanopore sequences were assembled *de novo* with Flye 2.9.1 (9). Illumina reads were aligned to the long-read assembly using Burrows-Wheeler aligner 0.7.17-r1198 (10) and polished with Pilon 1.24 (11) with option --fix all. Three rounds of polishing were conducted. Assembly quality was assessed with CheckM 1.0.18 (12). The assembly was annotated with the NCBI Prokaryotic Genome Annotation Pipeline 6.5 (13). Taxonomy was determined with GTDB-tk 2.2.5 (14). Computing resources were supplied and maintained by the Ohio Supercomputer Center (15) and the DOE Systems Biology Knowledgebase (KBase) (16). Default parameters were used unless otherwise specified.

The assembled genome consists of a 3.75 Mb circular chromosome and a 5.6 kb circular plasmid (Table 1). GTDB-tk identified *Halomonas* sp. GFAJ-1 (GCF_002966495.1, 97.33% average nucleotide identity) as the nearest neighbor. The genome encodes complete Embden-Meyerhof-Parnas, Entner-Doudoroff, tricarboxylic acid, glyoxylate, and pentose phosphate pathways. The genome lacks a canonical Complex I but encodes a Na^+^-translocating NADH-quinone oxidoreductase and all other components required for oxidative phosphorylation. Phosphate acetyltransferase (*pta*) and acetate kinase (a*ckA*) are identified in acetyl-CoA synthesis from acetate, supporting its ability to use acetate as an electron donor. All genes needed for dissimilatory nitrate reduction to ammonia are present (*napAB*, *nirBD*); however, M1 does not reduce nitrate *in vitro* (6). Sulfide:quinone oxidoreductase (*sqr*), sulfide dehydrogenase (*fccB*), thiosulfate dehydrogenase (*tsdA*), thiosulfate:cyanide sulfurtransferase (*tst*), and tetrathionate reductase subunit A (*ttrA*) are present and putatively involved in sulfur metabolism. Our genomic analysis of *Halomonas* sp. strain M1 provides a resource for continued study of the ecology of heterotrophic sulfur-oxidizing organisms in serpentinizing systems.

**TABLE 1:** Sequencing statistics for *Halomonas* sp. strain M1

Illumina reads accession no.	SRR23870724
Nanopore MinION reads accession no.	SRR23870723
Genome assembly accession no.	CP121119, CP121120
Number of total Illumina read pairs	2,123,948
Illumina assembly coverage	152x
miION estimated assembly coverage	163x
Number of contigs	2
Nanopore MinION read N_50_ ^a^	9,407
Assembly size (bp)	3,753,827
Number of protein-coding genes	3,354
Number of tRNA genes	60
Number of rRNAs (5S, 23S, 16S)	6, 6, 6
G+C content (%) for contig 1^b^	54.13
G+C content (%) for contig 2^b^	52.74
Estimated genome completeness (%)	100
Estimated contamination (%)	1.29

^a^ Determined with Flye 2.9.1.

^b^ Determined with CheckM v 1.0.18.

## Data Availability

The raw sequencing data were submitted to the NCBI Sequence Read Archive, and the genome assembly was submitted to GenBank under the accession numbers listed in Table 1 .
